# Carving out a Glycoside Hydrolase Active Site for Incorporation into a New Protein Scaffold Using Deep Network Hallucination

**DOI:** 10.1021/acssynbio.3c00674

**Published:** 2024-02-15

**Authors:** Anders Lønstrup Hansen, Frederik Friis Theisen, Ramon Crehuet, Enrique Marcos, Nushin Aghajari, Martin Willemoës

**Affiliations:** † The Linderstrøm-Lang Centre for Protein Science, Section for Biomolecular Sciences, Department of Biology, University of Copenhagen, Ole Maaløes Vej 5, 2200 Copenhagen, Denmark; ‡ Institute for Advanced Chemistry of Catalonia (IQAC), CSIC, Carrer Jordi Girona 18-26, 08034 Barcelona, Spain; § Protein Design and Modeling Lab, Department of Structural and Molecular Biology, Molecular Biology Institute of Barcelona (IBMB), CSIC, Baldiri Reixac 10, 08028 Barcelona, Spain; ∥ Molecular Microbiology and Structural Biochemistry, CNRS, University of Lyon1, UMR5086, 7 Passage du Vercors, F-69367 Lyon CEDEX 07, France

**Keywords:** de novo design, enzyme design, glycoside hydrolase, deep network hallucination

## Abstract

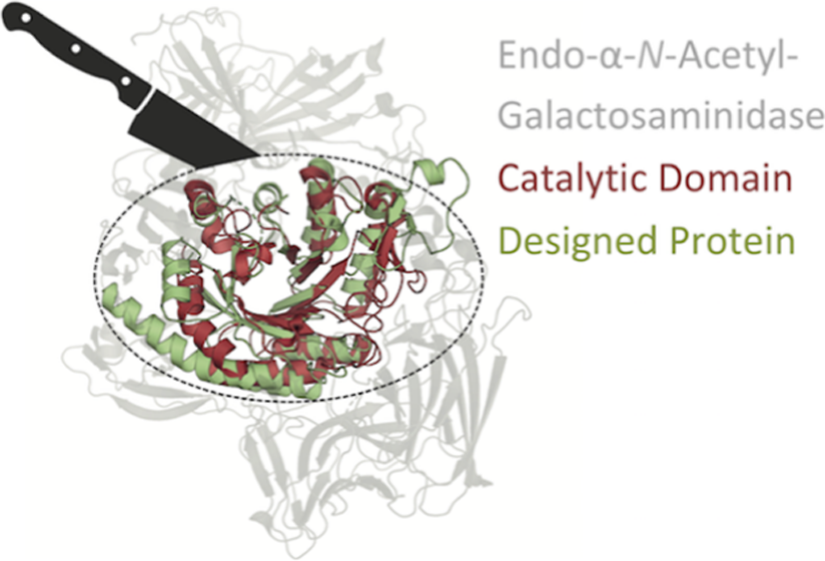

Enzymes are indispensable biocatalysts for numerous industrial applications, yet stability, selectivity, and restricted substrate recognition present limitations for their use. Despite the importance of enzyme engineering in overcoming these limitations, success is often challenged by the intricate architecture of enzymes derived from natural sources. Recent advances in computational methods have enabled the de novo design of simplified scaffolds with specific functional sites. Such scaffolds may be advantageous as platforms for enzyme engineering. Here, we present a strategy for the de novo design of a simplified scaffold of an endo-α-*N*-acetylgalactosaminidase active site, a glycoside hydrolase from the GH101 enzyme family. Using a combination of trRosetta hallucination, iterative cycles of deep-learning-based structure prediction, and ProteinMPNN sequence design, we designed proteins with 290 amino acids incorporating the active site while reducing the molecular weight by over 100 kDa compared to the initial endo-α-*N*-acetylgalactosaminidase. Of 11 tested designs, six were expressed as soluble monomers, displaying similar or increased thermostabilities compared to the natural enzyme. Despite lacking detectable enzymatic activity, the experimentally determined crystal structures of a representative design closely matched the design with a root-mean-square deviation of 1.0 Å, with most catalytically important side chains within 2.0 Å. The results highlight the potential of scaffold hallucination in designing proteins that may serve as a foundation for subsequent enzyme engineering.

## Introduction

Through evolution enzymes have been tuned to form intricate structures, particularly their active sites, which confer exceptional properties such as extreme rate accelerations and selectivity toward specific substrates.^1,2^ Enzymes may exhibit desirable catalytic properties for use in biomedical and industrial applications,^3,4^ but their sometimes complex, multidomain structures can pose significant challenges for both their practical application and engineering efforts aimed at optimizing their functionality.^5,6^

Enzyme scaffolds with tailored and less complex structures could be achieved through de novo design of small, stable proteins, recapitulating the desired active site geometry. Recently, deep learning-based hallucination approaches have been employed in de novo protein design through structure prediction networks such as trRosetta,^7^ AlphaFold2,^8^ and RoseTTAFold,^9^ allowing the search for amino acid sequences that are predicted to be well folded and satisfy a given structural design criteria.^10^ Hallucination approaches use algorithms that iteratively optimize an amino acid sequence based on a loss function typically related to a prediction of protein foldedness and structural recapitulation of a target functional motif.^11,12^ Introducing structural restraints allowed the design of proteins with specific structures but encoded by novel sequences. The combination of these two loss functions facilitated the design of protein sequences where selected regions satisfied specific structural restraints while surrounding regions would target any high-confidence prediction. This combined approach enabled de novo design of proteins with one or more specific functional motifs within an unspecified scaffold,^13,14^ thus introducing important versatility to the hallucination approach.

The endo-α-*N*-acetylgalactosaminidases (EC 3.2.1.97) of the GH101 enzyme family as classified in the CAZy database (www.cazy.org) catalyzes the hydrolysis of the mucin *O*-glycan, Galβ(1–3)GalNAc.^15,16^ Despite the more than 1350 amino acid residues large multidomain architecture of the GH101 family, the domain constituting the actual catalytic site is composed of only approximately 300 amino acids, forming a distorted (β/α)_8_ TIM-barrel-like fold sharing structural similarity with the GH13 α-amylase family.^17−19^ The substrate binding pocket of endo-α-*N*-acetylgalactosaminidases from *Streptococcus pneumoniae* (EngSP) and *Bifidobacterium longum* (EngBF) is shaped to complement the Galβ(1–3)GalNac substrate, whereas specific hydrogen bonds and local conformational changes involving a conserved tryptophan “lid” contribute to an occluded bound state.^17,18^ The anomeric carbon of Galβ(1–3)GalNac is positioned in close proximity to the catalytic nucleophile and acid/base that are central to the double-displacement mechanism retaining the stereochemistry of the glycan.^20−22^ The remaining structural domains presumably play crucial roles in maintaining overall enzyme stability and solubility or in providing functions such as macromolecular substrate recognition. Creating a molecule with a simplified scaffold and reduced molecular size would provide a more manageable GH101 template enzyme for engineering efforts aimed at processing and modifying complex glycans.

In the present study, we address current molecular size-related challenges in enzyme engineering of the EngBF active site by utilizing state-of-the-art methods for the de novo design of an enzyme scaffold with a significantly reduced size. To achieve this, we adapted the at that time available trRosetta hallucination methodology^10^ to generate novel sub-300 residue scaffolds capable of supporting the EngBF active site. Our results showed that trRosetta-hallucinated structures recapitulated the EngBF active site while reducing the molecular weight of the scaffold by more than 100 kDa. We then used the ProteinMPNN^23^ sequence design neural network to generate amino acid sequences that reliably encode the target structure. We found that iterative cycles of deep-learning-based structure prediction and ProteinMPNN sequence design (on the predicted backbone structures) progressively improved the designs by increasing the overall structure prediction confidence while keeping active site recapitulation and exploring a larger sequence space. Finally, we experimentally validated our design approach and found that six out of 11 designs were readily expressed as soluble monomers. Our designs exhibit a two-state unfolding reaction with similar or increased thermostability with respect to the native enzyme. One design was crystallized, allowing the structure to be determined, which closely matched the designed TIM-barrel fold, with an overall root-mean-square deviation (RMSD) of 1.0 Å for the core. It was found that a subset of the catalytically important residues identified in EngBF was situated in highly dynamic loops in the designed variants, which may contribute to the lack of detectable activity. However, the results indicate that it may be possible to hallucinate scaffolds presenting complex active sites.

## Materials and Methods

### trRosetta Hallucination Design

**Figure 1:**
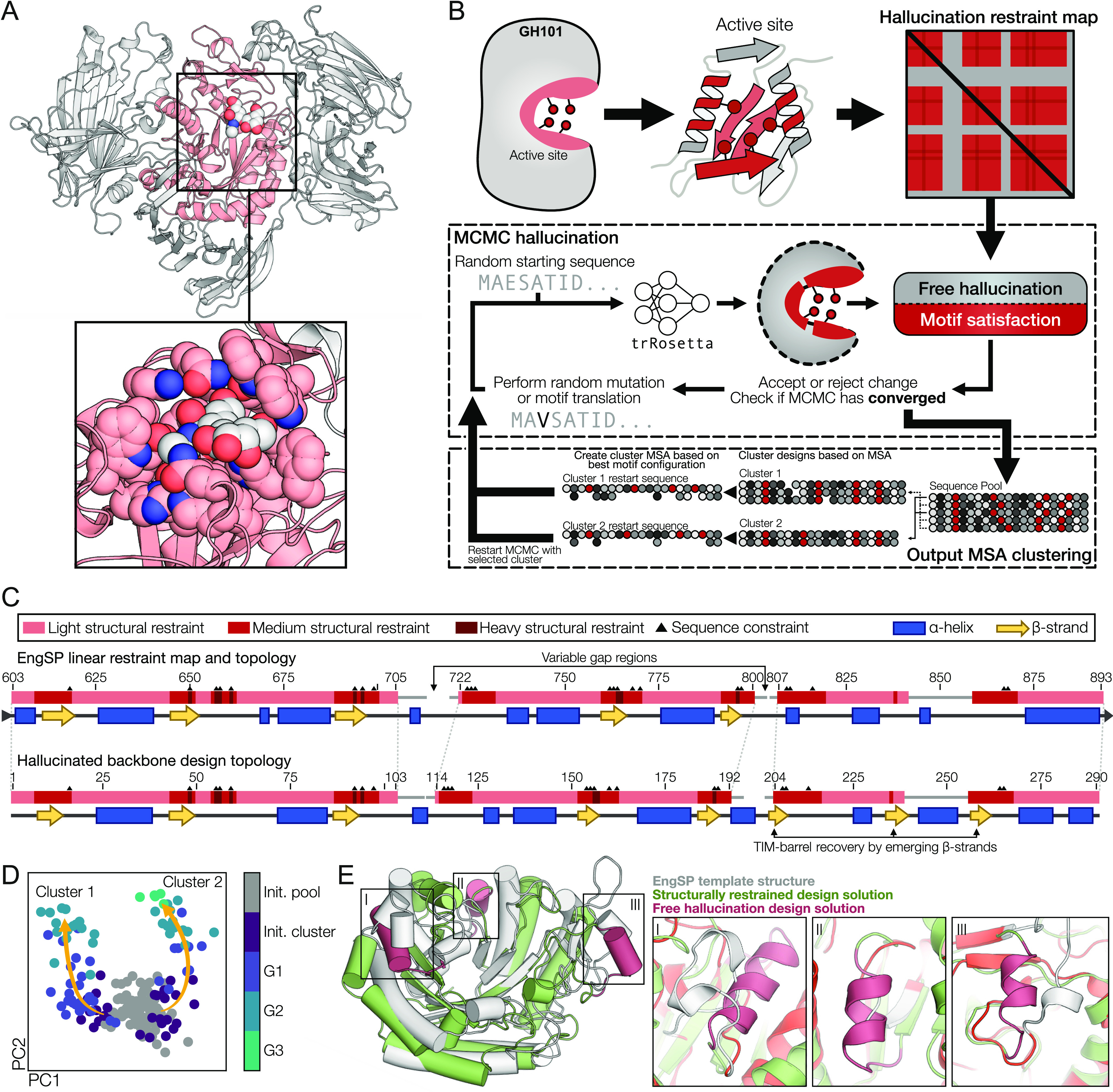
GH101 scaffold design using trRosetta hallucination and iterative sequence clustering. (A) Active site containing structure (red) of the EngSP (PDB 5A59) enzyme. The cutout shows the substrate bound in the active site with residues receiving a high restraint weight shown using atom spheres. (B) Scheme illustrating construction of the restraint map used to guide the trRosetta hallucination, the MCMC optimization, and the clustering step used to explore sequence- and motif-space. (C) Comparison of target (top) and design (bottom) topologies and restraint map. Yellow arrows indicate the β-strand, and blue boxes indicate the α-helix structure. Loss function weight of structural restraints is indicated by color with light, medium, and dark red representing low, medium, and high weight, respectively. Triangles indicate that the residue type was fixed to the WT type. (D) Principal component analysis of MSAs of all hallucinated sequences. The initial 136 sequences are labeled gray with the derived design generation following a color gradient. Yellow arrows illustrate the “evolutionary” path of the two selected clusters. (E) Example hallucinated design illustrating alternative solutions (red) to unrestrained regions superpositioned with the EngSP (PDB 5A59) catalytic domain.

We carried out deep network hallucination to design sequences optimizing a specified loss function using the trRosetta structure prediction network. To accommodate the strict structural requirements of enzyme active site dimensions, we adapted a publicly available trRosetta Markov chain Monte Carlo (MCMC) hallucination implementation^10^ to allow different weighting of positions related to the active site versus positions important for the overall fold. We employed three distinct weighting categories: low (weight of 1), medium (weight of 5), and heavy (weight of 10) applied as a linear map on the template protein sequence. Subsequently, we generated a two-dimensional restraint map from the linear maps, employing a multiplicative approach for the weights. In this scheme, the interaction between two heavily weighted residues is assigned a weight of 10 × 10 = 100, whereas interactions between two lightly weighted residues are assigned a minimal weight of 1 × 1 = 1. This approach allows for a nuanced modulation of restraint strengths based on their proximity to and influence on the enzyme’s active site. The structural restraints matching the EngSP crystal structure (PDB ID: 5A55) were implemented as discontinuous motifs with internal fixed-length gaps for short loop regions linking restrained structure (Figure 1). In addition, positions facing the active site were sequentially constrained to make sure the environment of the active site matched that observed in the crystal structure of the EngBF enzyme (PDB ID: 2ZXQ). At the beginning of each MCMC trajectory, the motifs would be placed randomly, but in their native order, within the design sequence. For each step, the MCMC algorithm would then perform either a random mutation of a nonsequentially constrained position or move a structural restraint, including potential sequential constraints within the motif.

Employing an iterative design methodology, we ensured convergence while navigating through a larger sequence space, thus mitigating the potential for prolonged searches in local loss function minima. This was implemented using a relatively short (5000 steps) MCMC optimization trajectory and thus obtaining a larger number of sequences. The output of each batch was then clustered according to their sequence similarity with the best cluster, based on overall prediction confidence, being used as the starting point for further MCMC optimization. In later iterations, the cluster multiple sequence alignment would be used to restrain the allowed mutations at specific positions. The hallucination process was run using a Pytorch implementation of trRosetta on a single Nvidia RTX3070 GPU, capable of performing 1.2 MCMC iterations per second on a 290-residue protein.

### ProteinMPNN Sequence Redesign

The hallucinated sequence of hEngBF2 was optimized by redesigning the structure model generated by OmegaFold^24^ using the ProteinMPNN sequence design neural network.^23^ To preserve the substrate binding site’s overall chemical properties, EngSP (PDB ID: 5A55) active site residues (Asp-658, His-661, His-694, Asn-696, Asn-764, Glu-796) in direct contact with the substrate were sequentially constrained. Initially, 232 sequences were generated with ProteinMPNN using a sampling temperature of 0.2, and sequence quality was assessed based on the OmegaFold model pLDDT and backbone recapitulation RMSD relative to the hEngBF2 model. Two sequences from the first round of sequence design were selected for further redesign, meeting the criteria of pLDDT > 75 and a backbone RMSD < 2 Å. Subsequent rounds of sequence redesign were conducted using the selected sequences’ OmegaFold models as the input backbone, generating and evaluating 100 sequences as described above. The selected designs’ pLDDT and scaffold recapitulation were validated against structure models independently predicted by ESMFold^25^ as an orthogonal test.

### Protein Synthesis and Purification

Synthesis and purification of the four dEngBF proteins were performed as follows: *Escherichia coli* BL21(DE3)^26^ cells transformed with pET11a-derived plasmids containing the reading frame of the 6xHis-tagged dEngBF of interest inserted between the NsiI and *Bam*HI restriction endonuclease sites of the vector were grown in 500 mL LB medium^27^ supplemented with 1% glucose and 0.2 mg/mL ampicillin and incubated while shaking at 37 °C until the optical density at 600 nm reached 0.6. Protein synthesis was then induced by the addition of isopropyl β-d-1-thiogalactopyranoside to a final concentration of 1 mM and continued for 4 h while shaking at 37 °C. Subsequently, the cells were harvested by centrifugation at 5000*g* at 4 °C for 20 min, and the cell pellets were stored at −20 °C. For protein purification, the cell pellets were resuspended in 20 mL of lysis buffer (20 mM sodium phosphate pH 7.4, 500 mM NaCl) at room temperature. The cell suspension was lysed by sonication (Hielscher UP200S) with alternating 30 s sonication bursts with 60 s rest periods (100% amplitude) for 10 min while on ice. Cell debris and insoluble material were removed by centrifugation at 20,000*g* at 4 °C for 20 min, and the supernatant was sterile filtered through a 0.45 μm syringe filter (Q-Max syringe filter 0.45 μm, CALS2504100S).

The dEngBF designs were purified using immobilized metal affinity chromatography on a 5 mL HisTrap FF column equilibrated in binding buffer (20 mM sodium phosphate, pH 7.4, 500 mM NaCl). To remove any unbound proteins, the column was washed with binding buffer until the absorbance at 280 nm of the eluate was stable. The dEngBF proteins were eluted with a linear gradient of 0 to 100% elution buffer (20 mM sodium phosphate, pH 7.4, 500 mM NaCl, 500 mM imidazole). The fractions representing the dEngBF were sterile filtered through a 0.45 μm syringe filter and dialyzed against 20 mM sodium phosphate pH 7.4, and 50 mM NaCl before storage at −20 °C. In a final purification step prior to biophysical characterization, the dEngBF eluate was loaded on a Superdex 200 (10/300) GL column equilibrated with the appropriate buffer for the experiment as outlined below.

The soluble fraction of the dEngBF protein yields was assessed by determining the total soluble yield per liter of culture equivalent, which was calculated by integrating the size exclusion chromatography elution profiles and normalizing by the sequence-specific extinction coefficients. The molar extinction coefficients and theoretical molecular weight were calculated from the candidate sequences using the ProtParam tool at the ExPASy Web site.^28^ The area under the curve of the size-exclusion chromatography profiles was determined by integrating the profiles using GraphPad Prism. Size exclusion chromatography analysis was performed by loading 0.5 mL of sample onto a Superose 12 (10/300) GL column, which was equilibrated with 10 mM sodium phosphate and 10 mM NaCl pH 7.4 and eluted with a flow rate of 0.5 mL/min. To assess the monomeric and soluble fraction of the design after heat treatment, samples were prepared by incubating 30 μM of the designs at 20 and 85 °C for 20 min. The absorbance of the size exclusion profiles before and after heat treatment was normalized to the maximum absorbance of the design elution peak at 20 °C.

### Circular Dichroism Spectroscopy

Circular dichroism (CD) experiments were conducted using a Jasco-J-815 spectrophotometer equipped with a Peltier-controlled cuvette holder. The designs were diluted to a concentration of 0.115 mg/mL in 10 mM sodium phosphate buffer at pH 7.4 and 10 mM NaCl. Spectra were acquired at 20 °C, above the melting temperature, and after cooling back to 20 °C between 260 and 195 nm with 10 scans accumulated using a data pitch of 0.2 nm, a path length of 0.1 cm, and a scan speed of 10 nm/min. The buffer spectrum was subtracted from the recorded spectra, and the reported measurements were within the instrument’s linear range. The mean residue weight ellipticity (deg cm^2^ dmol^–1^) was calculated using eq 1:
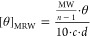
1where MW is the molecular weight (Da), *n* is the number of amino acid residues, θ is the observed signal (mdeg), *c* is the concentration (g/L), and *d* is the path length in cm.

For thermal melt experiments, the sample was heated between 20 and 90 °C at a rate of 1 °C/min, while measuring the signal at 222 nm. After it reached 90 °C, the sample was allowed to cool to 20 °C, and a spectrum was recorded to assess the reversibility of the unfolding. The thermal melting curves were fitted to eq 2:
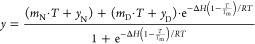
2where *y* is the observed signal, and *y*_N_ and *y*_D_ are baseline intercepts before and after the unfolding transition respectively, while *m*_N_ and *m*_D_ are baseline slopes before and after transition respectively. Δ*H*, is the van’t Hoff enthalpy at *T*_m_, *T*_m_ is the melting temperature, *T* is the temperature, and *R* is the gas constant.

### Differential Scanning Calorimetry

Differential scanning calorimetry experiments were performed on a MicroCal VP-DSC instrument at a temperature scan of 1 °C/min. All solutions were degassed, and the buffer spectra of 10 mM sodium phosphate buffer at pH 7.4 and 10 mM NaCl were recorded repeatedly until the instrument was stabilized. The sample cell was loaded with 0.5 mg/mL protein in 10 mM sodium phosphate, 10 mM NaCl, pH 7.4 and a temperature scan recorded within the range from 25 to 110 °C.

### Thermal Denaturation Using Nano Differential Scanning Fluorimetry

Two-dimensional denaturation of dEngBF proteins was followed using the Prometheus NT.48 (NanoTemper) recording fluorescence emission at 330 and 350 nm upon excitation at 280 nm. A denaturation dilution series was prepared by mixing different volumes of protein sample adjusted to a concentration of 15 μM with and without guanidine hydrochloride. The samples were loaded into Prometheus NT.48 high sensitivity capillaries (NanoTemper technologies cat. no. PR-C006) and sealed with high vacuum grease (Dow Corning) to avoid evaporation during the experiment. The excitation power was set to ensure that the fluorescence signal was within the linear range of the instrument. The temperature range was 20–95 °C with a temperature increment of 1 °C/min. For each candidate, the melting curves for each concentration of denaturant were fitted to the following eq 3 correcting for pre- and post-transition by adding quadratic terms:^29^
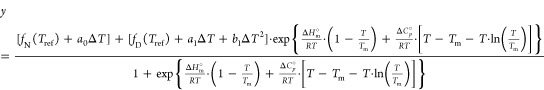
3where  is the change in enthalpy upon unfolding at *T*_m_, ΔC_p_^o^ is the change in the heat capacity of the system assumed to be independent of temperature.^30^*T*_m_ is the melting temperature and *R* is the gas constant while the constants *a*_0_, *a*_1_, and *b*_1_ are constants describing the temperature dependence of the fluorescence signal. Δ*T* is defined as Δ*T* = *T* – *T*_ref_ where *T*_ref_ is an arbitrary reference temperature.

### Molecular Dynamics Simulations

Molecular dynamics simulations of dEngBF models were performed to evaluate the stability, alleviate potential structural strains arising from the design, and investigate the dynamics and flexibility of distinct regions within the dEngBF models. As the dEngBF models were predicted to exhibit monomeric structures without cofactors, a standardized setup procedure was followed. Initially, hydrogen atoms were added to the structures using standard protonation states for all residues. Subsequently, the structures were solvated in a dodecahedral box with a minimum distance of 1 nm from the protein atoms. Sodium chloride ions were introduced to achieve a salt concentration of 100 mM. Hydrogen and water atoms were equilibrated in a 2 ns restrained *NVT* simulation followed by a 2 ns *NPT* simulation with restraints on the protein heavy atoms. Following equilibration, production simulations were conducted for a duration of 1 μs for all proteins except for the dEngBF4 model, which was simulated for 2 μs. The initial 200 ns of each simulation was designated as an equilibration phase and excluded from subsequent statistical analyses. A time step of 4 fs was achieved through the implementation of virtual sites on the hydrogen atoms. The simulations employed the amber99SB-disp force field,^31^ which has demonstrated accuracy in representing the structure and dynamics of both ordered and disordered regions.^32,33^ Electrostatic interactions were computed using the Particle Mesh Ewald algorithm, employing a 1 nm cutoff. Temperature coupling utilized the V-rescale algorithm, maintaining a temperature of 310 K, while pressure coupling was done via the Parrinello–Rahman algorithm at a pressure of 1 atm. Constraints were applied to hydrogen bonds, and the modified TIP4P water model, accompanying the amber99SB-disp force field, was employed. All simulations and setup procedures were conducted using the Gromacs software,^34,35^ and trajectory analyses were performed utilizing the MDtraj Python library.^36^ Calculation of the average structure and root-mean square fluctuations (RMSF) was executed utilizing the Bayesian-based Theseus method.^37^

### Crystallization of dEngBF4

Crystallization conditions screening was carried out at 292 K using commercially available crystallization kits and employing the vapor-diffusion method in sitting drops. For this purpose, a Mosquito crystallization robot from STP Labtech was employed using two protein/crystallization agent ratios (200 + 100 and 100 + 100 nL drops), respectively, equilibrated against 70 μL in MRC crystallization plates (Molecular Dimensions). dEngBF4 was concentrated to 7 mg mL^–1^ in a 10 mM tris-HCl pH 8.0, 10 mM NaCl buffer, and crystallized under two different conditions (hereafter form 1 and 2). Crystals of form 1 were obtained under two different conditions in drops containing 200 nL of protein + 100 nL of well solution, where the latter was 0.15 M ammonium nitrate, 20% (v/v) PEG SM, 5% (w/v) ethylene glycol, and 0.1 M MES pH 6.0 (form 1a) and 25% (v/v) PEG SM and 0.1 M CBTP pH 5.5- (form 1b). Those of form 2 were grown from a solution containing 18% (w/v) PEG SM, 10% (w/v) ethylene glycol, 0.10 M MgSO_4_, and 0.1 M KCl in a 100 nL protein + 100 nL well solution ratio.

### Crystal Structure Determination

X-ray diffraction data were collected on beamlines ID30B and ID23-2 at the ESRF in Grenoble (France). All data were reduced using the autoprocessing procedure at the ESRF “GrenADES” involving programs from the XDS package,^38^ the CCP4 suite,^39^ and STARANISO.^40^ A first data set of form 1b collected on ID23-2 (wavelength 0.8731 Å) to 2.37 Å resolution was used for partial structure determination in a molecular replacement search using the OmegaFold model of dEngBF4 and the MoRDa molecular replacement pipeline.^41^ The model solution displayed a *Q* factor of 0.78. This partial initial model was built and corrected manually using COOT^42^ interspersed with maximum likelihood refinement using the program REFMAC V.5.8.^43^ A second set of data was collected on form 1a to 1.94 Å resolution, and the corresponding structure was solved by molecular replacement using molecule A of the form 1b structure as a search model. The solution had a *Q* factor of 0.825. Finally, the form 2 structure of dEngBF4 was solved from data collected to 3.08 Å resolution by molecular replacement using molecule A from the refined structure of form 1a as the search model, giving rise to a *Q* factor of 0.885. These two latter data sets were collected on the ID30B beamline (ESRF, Grenoble), and the MoRDa molecular replacement pipeline was used for both structure determinations. Manual iterative rebuilding and fitting were done using COOT and model refinement was done with REFMAC V.5.8^41^ using restrained refinement.^44^ Model geometry and stereochemistry were examined using Molprobity.^45^

### dEngBF Activity Assays with Galβ(1–3)GalNAcα1-*para*-Nitrophenol

Activity assays for dEngBF were conducted using Galβ(1–3)GalNAcα1-*para*-nitrophenol as the substrate. The assays were carried out in a reaction mixture containing 5 mM substrate, 50 mM sodium acetate, and 50 mM NaCl, at pH 5.0 and 6.0. The dEngBF protein concentrations were adjusted to a final concentration of 20 μM and incubated at 37 °C for 24 h. To quench the reactions, 150 μL of the reaction mixture was transferred to a 96-well microtiter plate containing 150 μL of 1 M sodium carbonate, maintained on ice to minimize evaporation. The subsequent quantification of the released *para*-nitrophenol was conducted in a plate reader by measuring the absorbance at 410 nm.

### dEngBF Interaction with Galβ(1–3)GalNAcα1-*para*-Nitrophenol Substrate

Differential scanning calorimetry experiments were performed on a MicroCal VP-DSC instrument in the absence or presence of 2 mM Galβ(1–3)GalNAcα1-*para*-nitrophenol substrate. Initially, degassed buffer (10 mM sodium phosphate, pH 7.4, 10 mM NaCl) in both the reference and sample cell allowed for the instrument to stabilize at a temperature scan of 1 °C/min. Protein (0.5 mg/mL protein in the same buffer) was then loaded into the sample cell and a temperature scan was recorded from 25 to 90 °C.

## Results

### Minimal GH101 Scaffolds Generated via Deep Network Hallucination

The first step in reducing the size of the GH101 enzyme members was to obtain a backbone structure compatible with the scaffolding of the active site. The enzymes of the GH101 family are composed of a multidomain structure consisting of over 1000 amino acid residues. The active site, here defined as all the residues located within 15 Å of the bound substrate (PDB ID: 5A59), is mainly located within a 300-residue domain that is embedded in the overall enzyme structure (Figure 1A). To design a scaffold much reduced in size compared to GH101 members and potentially encompassing a functional GH101 active site, we exploited recent advances in protein design using deep learning.^10^ This was done with an MCMC hallucination approach using the trRosetta deep neural network^7^ (Figure 1B). The hallucination approach was guided by a loss function composed of one component to favor folded protein structures and a second component to recapitulate the desired active site geometry. The scaffold accommodating the GH101 active site structure (Figure 1A) was obtained by applying restraints to the structural elements defining the active site and residues in contact with the substrate. The EngSP (PDB ID: 5A59) structure was used as a template to create a restraint map describing three discontinuous motifs, spanning residues 603–705, 722–800, and 807–893. Most residues were given a low weight, while residues defining the core β-strands were generally given a medium weight, and residues contacting the substrate were given a high weight in addition to being constrained sequentially (Figure 1C). In total, 23 residues were sequentially constrained. To allow for unrestrained regions to adopt folded structures, the above loss function incorporated a “free hallucination” component that maximized the Kullback–Leibler divergence, with respect to a random sequence background, of unrestrained residues.

**Figure 2:**
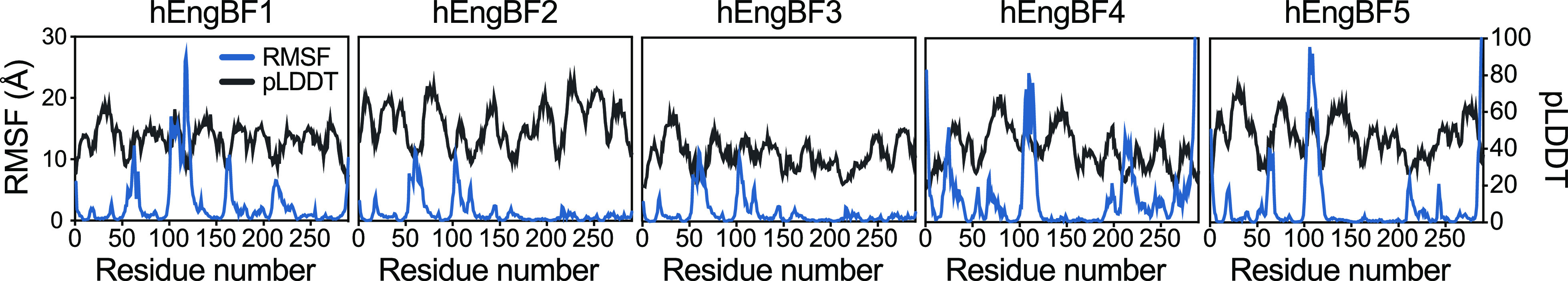
RMSF (blue) of selected hEngBF hallucinated designs Cα atoms sampled by molecular dynamics simulation and the pLDDT (black) from the OmegaFold predicted structural models plotted as a function of the residue number.

Using the hallucination approach described above, we generated 136 sequences, designated hEngBFs, that were clustered according to their sequence similarity. Based on the loss function scores, two clusters were selected for further sequence optimization by employing an iterative approach using a consensus sequence from multiple sequence alignments of the clusters. These clusters were individually used as starting sequences for further hallucination as described in Materials and Methods (Figure 1B). The two sequentially distinct solutions scaffolding the GH101 active site (Figure 1D) showed no significant homology to existing proteins based on BLAST analysis. These final designs encompassed the structural features of the target fold of the EngSP active site very well (RMSD < 1.0 Å), with unrestrained regions that adopted different structural solutions to maximize the prediction confidence (Figure 1E). Despite all the hallucinated sequences being generally predicted to achieve the desired fold, the prediction confidence was low for all designs (mean OmegaFold pLDDT < 53). Five designs, all adopting a TIM-barrel fold, were selected based on their alignment with the EngSP active site and the positioning of catalytic residues. As an orthogonal test, molecular dynamics simulations served to assess the structural rigidity of the hallucinated backbone geometry (as a proxy of folding stability) and revealed one design (hEngBF2, Figure 2) with considerably lower RMSF, indicating a more favorable specific backbone geometry. The hEngBF2 design was therefore selected as a platform for further optimization.

### Model Confidence Improved through Sequence Redesign of Designed EngSP Active Site Scaffolds

**Figure 3:**
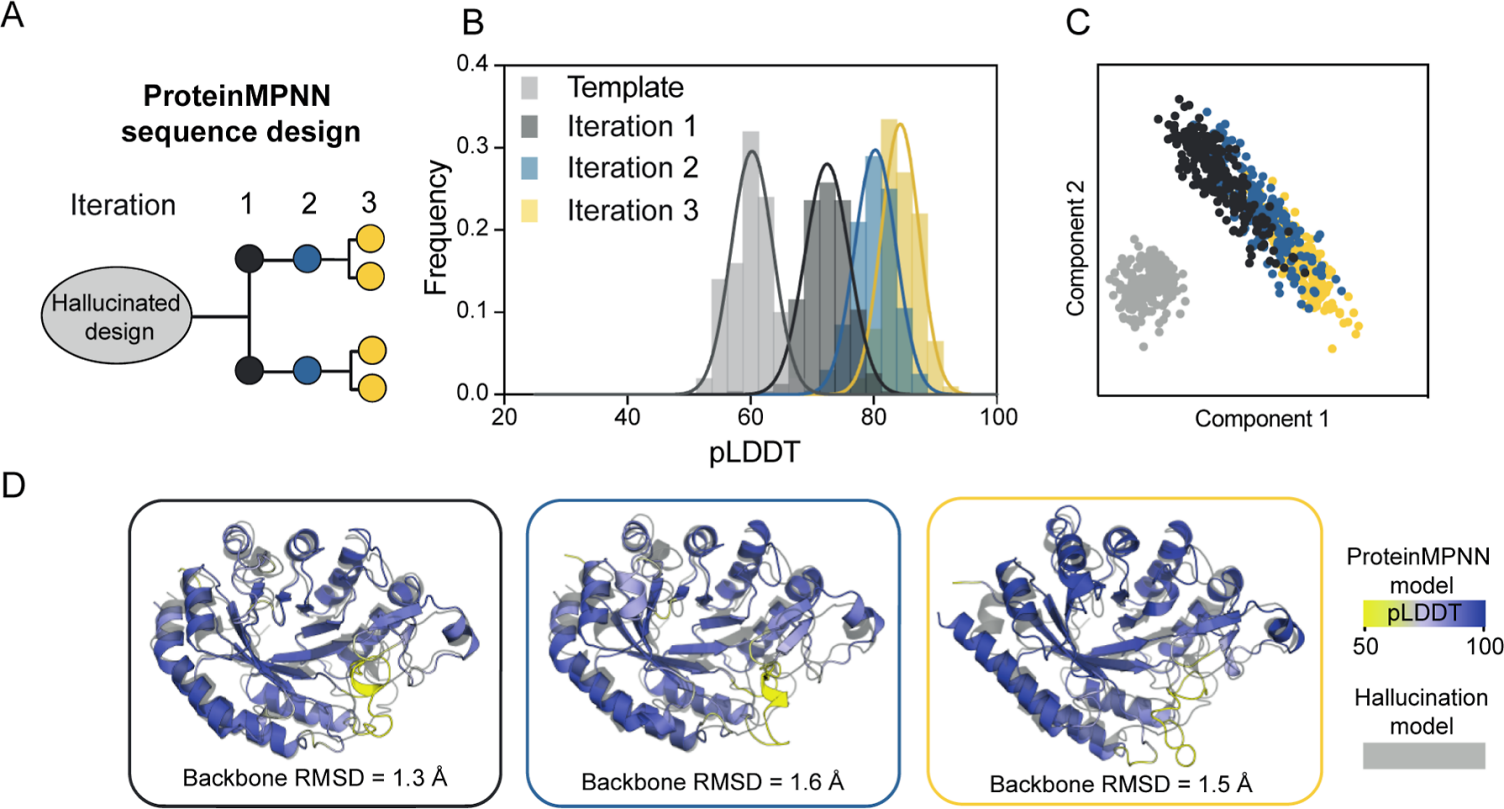
ProteinMPNN sequence redesign. (A) Schematic representation of the iterative sequence redesign using the ProteinMPNN algorithm. Starting from the hEngBF2 hallucinated design model successive rounds and evaluation of the ProteinMPNN output was performed. (B) Histograms of pLDDT scores from OmegaFold structure predictions of hallucinated and ProteinMPNN redesigned sequences. Each histogram represents the distribution of pLDDT scores for a given round with mean values of 60 for the hallucinated sequence and 72, 80, and 84 for iterations 1 –3 of the ProteinMPNN sequence redesign, respectively. (C) Principal component analysis of MSAs of all hallucinated and ProteinMPNN redesigned sequences. Each point on the plot represents a sequence, and the distance between points indicates how similar the sequences are. The color of each point indicates the iteration of sequence redesign (light gray: hallucinated sequences, dark gray: iteration 1, Blue: iteration 2, yellow: iteration 3). (D) OmegaFold structure prediction of representative sequences from iteration 1 (left panel), 2 (middle panel), and 3 (right panel) of the ProteinMPNN sequence redesign, superimposed with the OmegaFold structural model of hEngBF2 (gray). The pLDDT prediction confidence scale is indicated on the structure, ranging from 50 (yellow) to 100 (blue). The RMSD is for the predicted structure of the representative sequence with respect to the hEngBF2 model.

The initial sequence designs generated through the hallucination approach had low prediction confidence but showed a reasonable overlay of the catalytic site. To improve the prediction confidence, an iterative protein sequence redesign approach utilizing ProteinMPNN (Figure 3A) was employed. Analogous to the hallucination process, important side chains in the active site were constrained and, based on hEngBF2 as a structural template (Figure 2), a set of 232 sequences were generated with structural models predicted using OmegaFold. The structural models were ranked based on the pLDDT confidence scores and inspected manually to evaluate the active site geometry in comparison to the native enzyme. The two top-ranking models from each round of sequence redesign were used as a starting point for subsequent rounds. Over three iterations, starting from the best sequence of each round, the mean pLDDT confidence score was improved, indicating a progressive enhancement in the overall scaffold quality (Figure 3A,B). Analysis of the redesigned sequences revealed that the ProteinMPNN algorithm efficiently explored a region of sequence space that trRosetta alone was unable to access (Figure 3C), while maintaining a similar (RMSD < 1.6 Å) active site structure (Figure 3D). This could likely be attributed to the predictions by OmegaFold, providing ProteinMPNN with a better backbone and allowing improved sequence redesign. Although the pLDDT confidence score improved, some regions retained their low confidence potentially due to poor scaffold geometry (Figure 3D). Overall, the combination of backbone scaffold hallucination and sequence redesign demonstrated the potential to efficiently produce sequences that strongly encode the desired structure while allowing the exploration of previously unexplored sequence space.

Molecular dynamics simulations report an overall rigidification of the enzyme along successive design iterations (Figure 3) as residue fluctuations progressively reduce (Figure S2). However, this does not correlate with a higher rigidification of active site residues, which does not show any clear trend. It is still an open question the amount of flexibility needed in active enzymes and how it should be shared by different protein regions,^46^ but it is clear that current design algorithms are unable to encode or select for this feature, which is crucial for enzyme catalysis.

### Expression of *de novo* Designs Reveals High-Yield Monomeric Proteins

**Figure 4:**
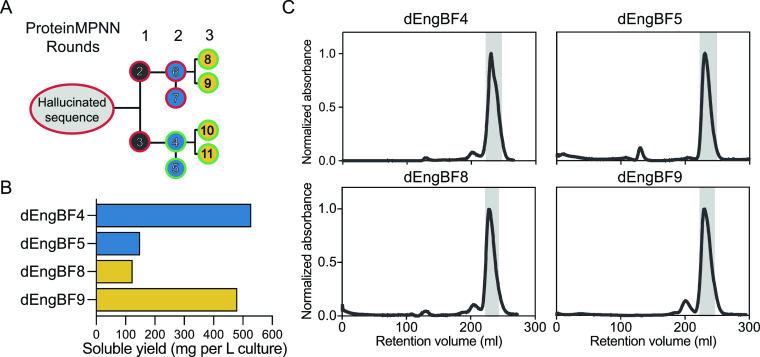
Experimental validation of hallucinated and ProteinMPNN redesigned sequences. (A) Schematic of the sequence design process, starting with the hallucinated sequence and followed by a ProteinMPNN sequence redesign. The 11 designed sequences are labeled 1–11 and color-coded based on the round of redesign, ranging from iteration 1–3. The edges of the circles are color coded based on protein expression, with green indicating detectable protein expression and red indicating no detectable protein expression. (B) Quantification of total soluble yield per liter of culture for purified protein designs from size exclusion chromatography elution profiles. (C) Size-exclusion chromatography profiles of purified dEngBF4, dEngBF5, dEngBF8, and dEngBF9, eluted in 10 mM sodium phosphate pH 7.4, 10 mM NaCl using a Superdex 200 26/60 column. Gray shading represents the elution profile and the fractions collected.

Ten sequences, exhibiting a sequence identity with the native enzyme domain within the range of 13–16% was selected from the pool of designs generated through the combined hallucination process and ProteinMPNN sequence redesign. Samples from cells expressing the reading frames corresponding to the original hallucinated sequence and the two sequences from the first round of ProteinMPNN redesign did not exhibit readily detectable levels of protein synthesis. In contrast, cells expressing the corresponding reading frames of six out of eight sequences derived from round two and three of the ProteinMPNN redesign procedure (Figure 1A), produced SDS-PAGE detectable levels of protein with the expected molecular weight confirmed through SDS-PAGE analysis (data not shown). Among these, dEngBF4 and dEngBF9 gave high protein yields (∼500 mg/L culture), while dEngBF5 and dEngBF8 produced a little less (∼100 mg/L culture) (Figure 4B). When analyzed under native conditions using size exclusion chromatography, the oligomeric state and purity of these four proteins were assessed and all displayed a single elution peak, consistent with the expected behavior of a monomeric globular protein (Figure 4C).

### dEngBF Proteins Show High Thermal Stability

**Figure 5:**
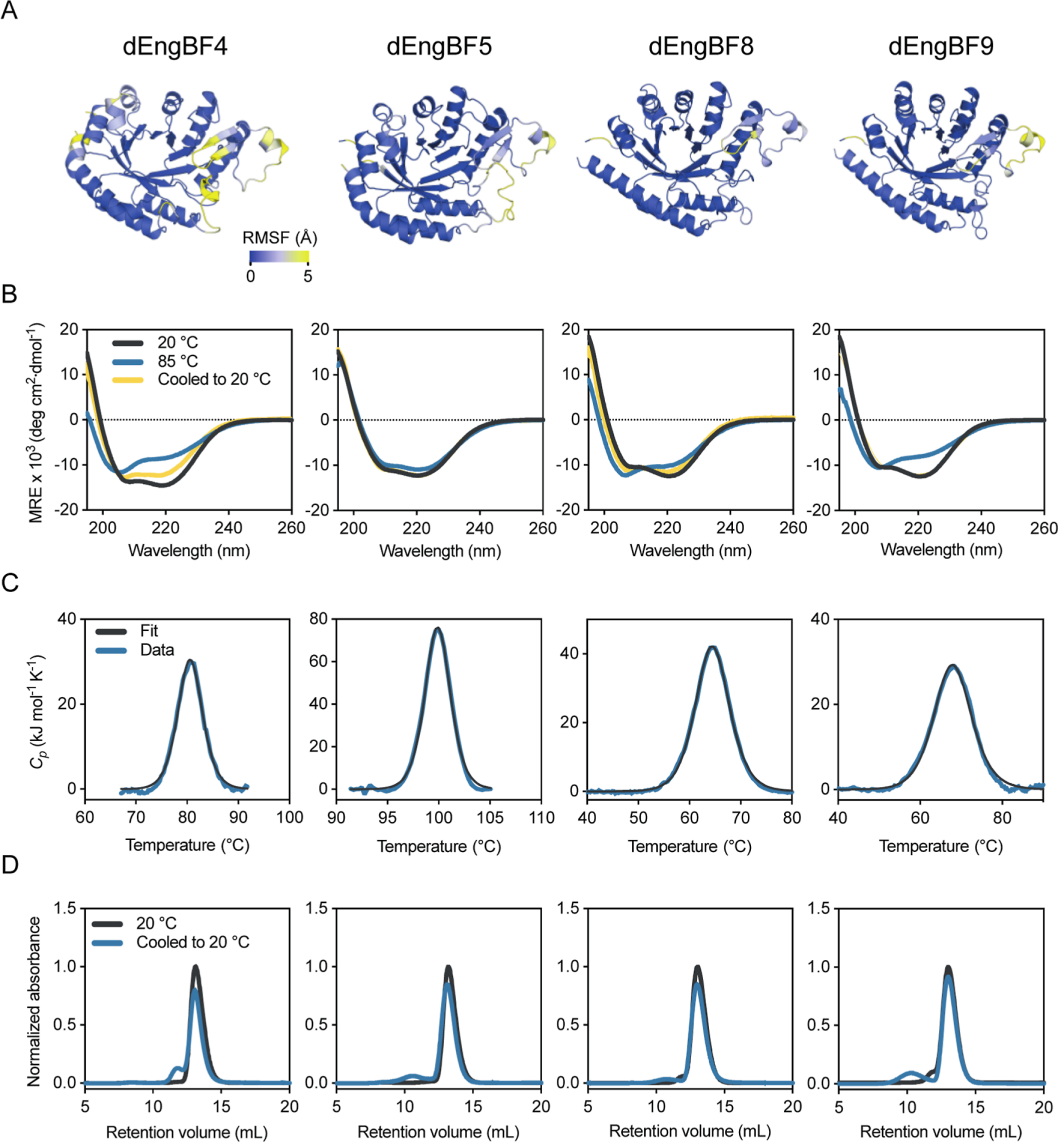
Biophysical and in silico characterizations of dEngBF designs. The name of the designed protein for which data are presented in panels A–D is listed on top. (A) RMSF of dEngBF models Cα atoms sampled by molecular dynamics simulation and plotted on the OmegaFold predicted structural models. (B) Far-UV CD spectroscopy of dEngBF designs at 20 °C (dark gray), 85 °C (blue), and cooled back to 20 °C (yellow). MRE is the mean residue ellipticity (eq 1). (C) Differential scanning calorimetry thermograms of dEngBF design unfolding. The thermograms show the unfolding of the designed proteins as a function of temperature. (D) Size-exclusion chromatography profiles of dEngBF designs at 20 °C before (dark gray) and after (blue) thermal unfolding.

To assess the dynamics and stability of the designed proteins, all-atom molecular dynamics simulations were performed. The simulations revealed a remarkably rigid TIM-barrel core with loop regions that showed a high level of dynamics with RMSF values ranging between 5 and 10 Å for dEngBF4, dEngBF5, and dEngBF9 (Figure 5A). Notably, dEngBF8 appeared more rigid, with lower loop RMSF values, and RMSF values above 5 Å were observed only in the C- and N-terminal regions, indicating the efficacy of the scaffold in conferring stability to the core structure. That high RMSF values correlated with low pLDDT scores indicate a potential for further optimization of the structural stability of the designs.

**Figure 6:**
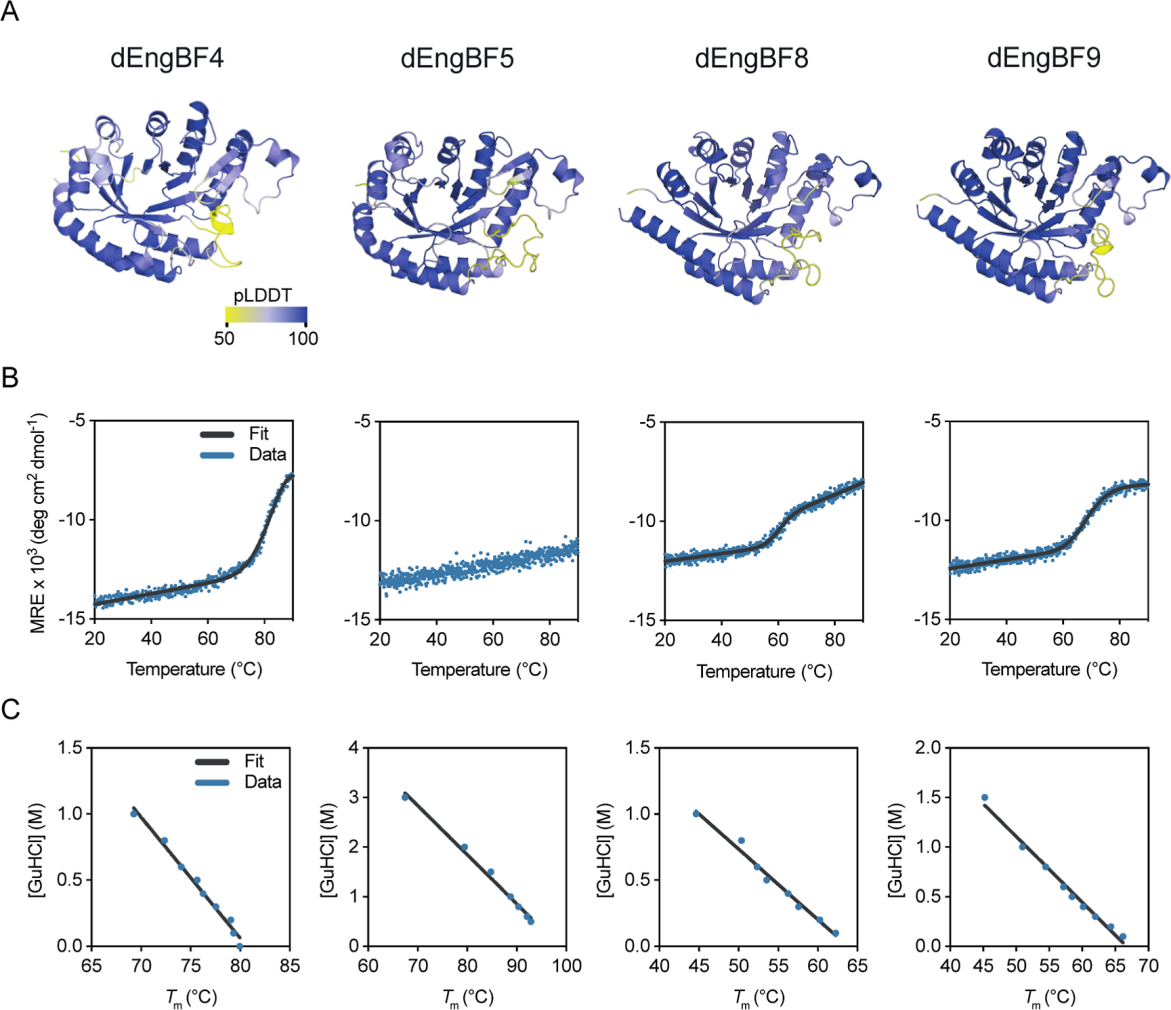
(A) OmegaFold pLDDT prediction confidence plotted on the structures of dEngBF designs. (B) CD spectroscopy thermal denaturation of dEngBF designs followed at 280 nm. (C) The denaturant concentration was plotted as a function of the design melting temperature. The *T*_m_ at [GuHCl] = 0 is extrapolated from a linear fit.

Far-UV CD spectra recorded at 20 °C showed features characteristic of well-folded proteins with both α-helix and β-sheet secondary structures (Figure 5B). Analysis of the thermostability of the proteins revealed large differences in their thermal stability. Specifically, compared to EngBF, dEngBF4 and dEngBF5 showed higher thermal stability with *T*_m_ values of 81.1 ± 0.6 and 99.9 ± 0.1 °C, respectively (Figure 5C and Table 1). While both dEngBF4 and dEngBF5 exhibited high thermal stability, their differential scanning calorimetry thermograms when analyzed indicated that the unfolding process under the used conditions was non-two-state and from repeated heating cycles proved irreversible (Figures 5C and S1). In contrast, the thermograms of dEngBF8 and dEngBF9 showed reversible two-state unfolding and *T*_m_ values comparable to EngBF. The *T*_m_ values of the proteins were confirmed by CD spectroscopy and nano differential scanning fluorimetry (Figure 6 and Table 2). CD spectra of the proteins following heat treatment and subsequent cooling showed a significant loss of secondary structure at temperatures above *T*_m_ and recovery of most of the secondary structure upon cooling to 20 °C (Figure 5B). Size-exclusion chromatography of the same samples gave elution profiles (Figure 5D) indicating that the proteins regained their globular structure upon cooling.

**Table 1:** Differential Scanning Calorimetry Derived Thermodynamic Parameters of Unfolding of dEngBF Proteins Including EngBF^a^

protein	no. of residues	*T*_m_ (°C)^b^	Δ*H*_m_ (kJ/mol)^b^
dEngBF4	300	81.1 ± 0.6	193 ± 2
dEngBF5	300	99.9 ± 0.1	251 ± 2
dEngBF8^c^	300	64.5 ± 0.1	410 ± 30
dEngBF9^c^	300	68.0 ± 0.1	356 ± 3
EngBF	1363	68.2 ± 0.1	1120 ± 20

a Designed proteins include a 6xHis tag and a four-residue linker.

b Standard errors are derived from the fit.

c Reversible unfolding with no detectable aggregation.

**Table 2:** CD Spectroscopy and Nano Differential Scanning Fluorimetry Derived Melting Temperatures^a^

protein	no. of residues	CD *T*_m_ (°C)^b^	nanoDSF extrapolated *T*_m_ (°C)^b^
dEngBF4	300	85.7 ± 8.1	80.7 ± 0.7
dEngBF5	300	ND^c^	98.7 ± 1.9
dEngBF8^d^	300	62.9 ± 0.1	62.3 ± 0.6
dEngBF9^d^	300	68.6 ± 0.6	66.8 ± 1.2
EngBF WT	1363	68.9 ± 1.2	ND^c^

a Nano differential scanning fluorimetry melting temperatures are obtained from extrapolation to [GuHCl] = 0 as shown in Figure 6. Designed proteins include a 6xHis tag and a four-residue linker.

b Standard errors are derived from the fit.

c Not determined.

d Reversible unfolding with no detectable aggregation.

### dEngBF4 Crystal Structure Reveals a (β/α)_8_ Barrel Fold with Dynamic Loops

**Figure 7:**
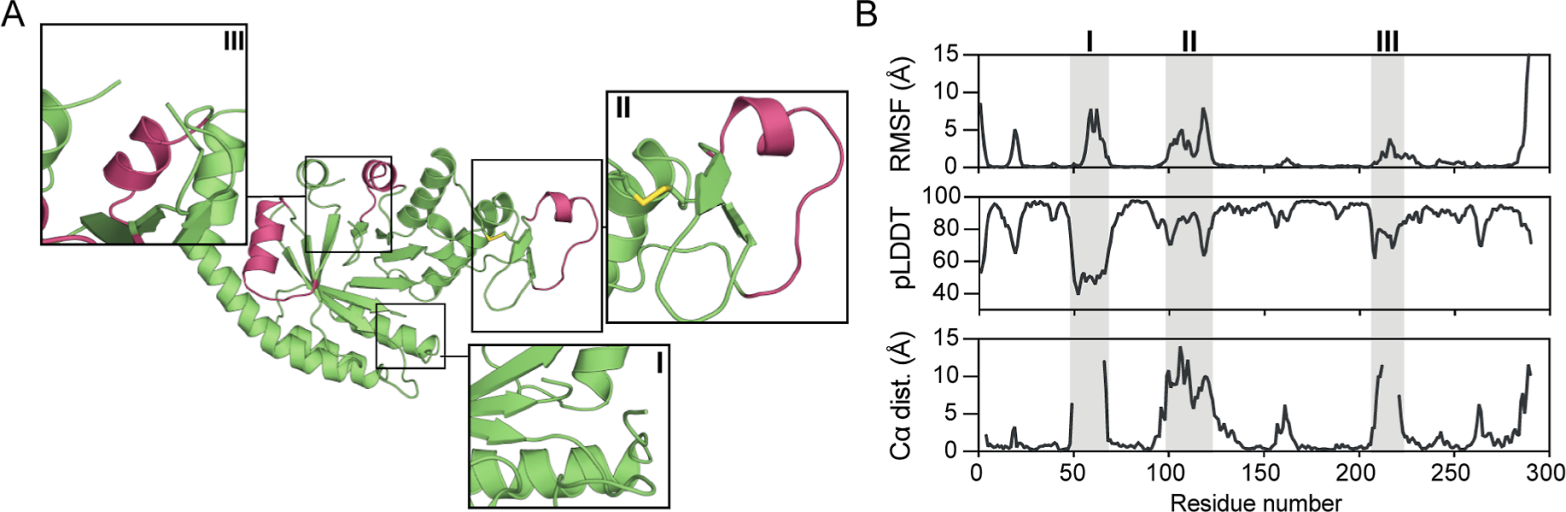
(A) Structure of dEngBF4 solved by X-ray crystallography (molecule A of PDB: 8QYE). Unrestrained segments during the design phase are depicted in red. Zoomed insets emphasize areas that either displayed limited electron density in the crystal structure or exhibited pronounced dynamics in molecular dynamics simulations. (B) Plots depicting dEngBF4 per-residue Cα RMSF sampled from molecular dynamics simulations (top panel), OmegaFold pLDDT prediction confidence (middle panel), and distances between experimental and structural model Cα atoms (bottom panel).

dEngBF4 crystallized in two different space groups, *P*2_1_2_1_2_1_ (form 1) and *P*6_1_ (form 2), and the crystal structures of the protein were determined using the molecular replacement method described in Materials and Methods. Data collection and refinement statistics are listed in Table 3. Form 1 has two protein molecules, 1A and 1B, in the asymmetric unit, whereas for form 2 one protein molecule is present in the asymmetric unit, resulting in three separate structures. All three structures aligned with an RMSD of 1.0 Å or less, revealing a common (β/α)_8_-barrel organization with an overall fold similar to the hallucinated model (Figure 7A). For molecule 1A, more residues were observed in the electron density than in the two other molecules (see above), with the lacking electron density mostly corresponding to residues present within loop regions. Specifically, loop regions ranging from Gly50-Gly65 and Glu210-Val220 were poorly defined or lacked electron density (Figure 7A, insert I and II), correlating with regions displaying increased dynamics in the molecular dynamics simulations and low pLDDT confidence score (Figure 7B). Likewise, another extended loop region including His91-Lys127 harboring the two tryptophan residues, making up the tryptophan lid in EngBF, displayed both low pLDDT confidence scores and flexibility in the molecular dynamics simulation. The movement of this loop, however, is constrained due to the formation of a disulfide bond between Cys124 and Cys163 residues, potentially contributing to the observed high thermostability in dEngBF4. Interestingly, in contrast, dEngBF5, dEngBF8, and dEngBF9 feature either only one or no cysteines in their sequence, respectively.

**Table 3:** Data Collection and Refinement Statistics for dEngBF4 Crystal Structures^a^

crystal	form 1	form 2
PDB ID	8QYE	8QZK
data collection		
beamline	ID30B	ID30B
wavelength (Å)	0.8731	0.8731
resolution range (Å)	66.87–2.05 (2.12–2.05)	51.95–3.08 (3.16–3.08)
space group	*P*2_1_2_1_2_1_	*P*6_1_
*a*, *b*, *c* (Å)	35.87, 70.49, 197.25	109.54, 109.54, 62.09
α, β, γ (deg)	90, 90, 90	90, 90, 120
unique reflections	32,254 (2194)	14,461 (2640)
multiplicity	4.4 (4.4)	2.4 (2.4)
completeness (%)	99.3 (97.6)	97.7 (97.7)
mean *I*/σ(*I*)	13.4 (1.6)	8.5 (1.3)
Rmerge	0.07 (1.11)	0.072 (0.804)
CC1/2	0.99 (0. 53)	0.99 (0.50)
refinement		
protein atoms	3623	1883
solvent atoms	195	24
Rwork	0.2285	0.2311
Rfree	0.2730	0.2748
RMSD bonds (Å)	0.003	0.003
RMSD angles (deg)	0.545	0.563
average *B*-factor (Å^2^)	46.58	101.55
Ramachandran plot		
favored (%)	95.11	90.07
allowed (%)	3.40	5.53
outliers (%)	1.49	3.40

a Values in parentheses are for the highest resolution shell.

### Crystal Structure Shows Good Agreement with Hallucinated Backbone

**Figure 8:**
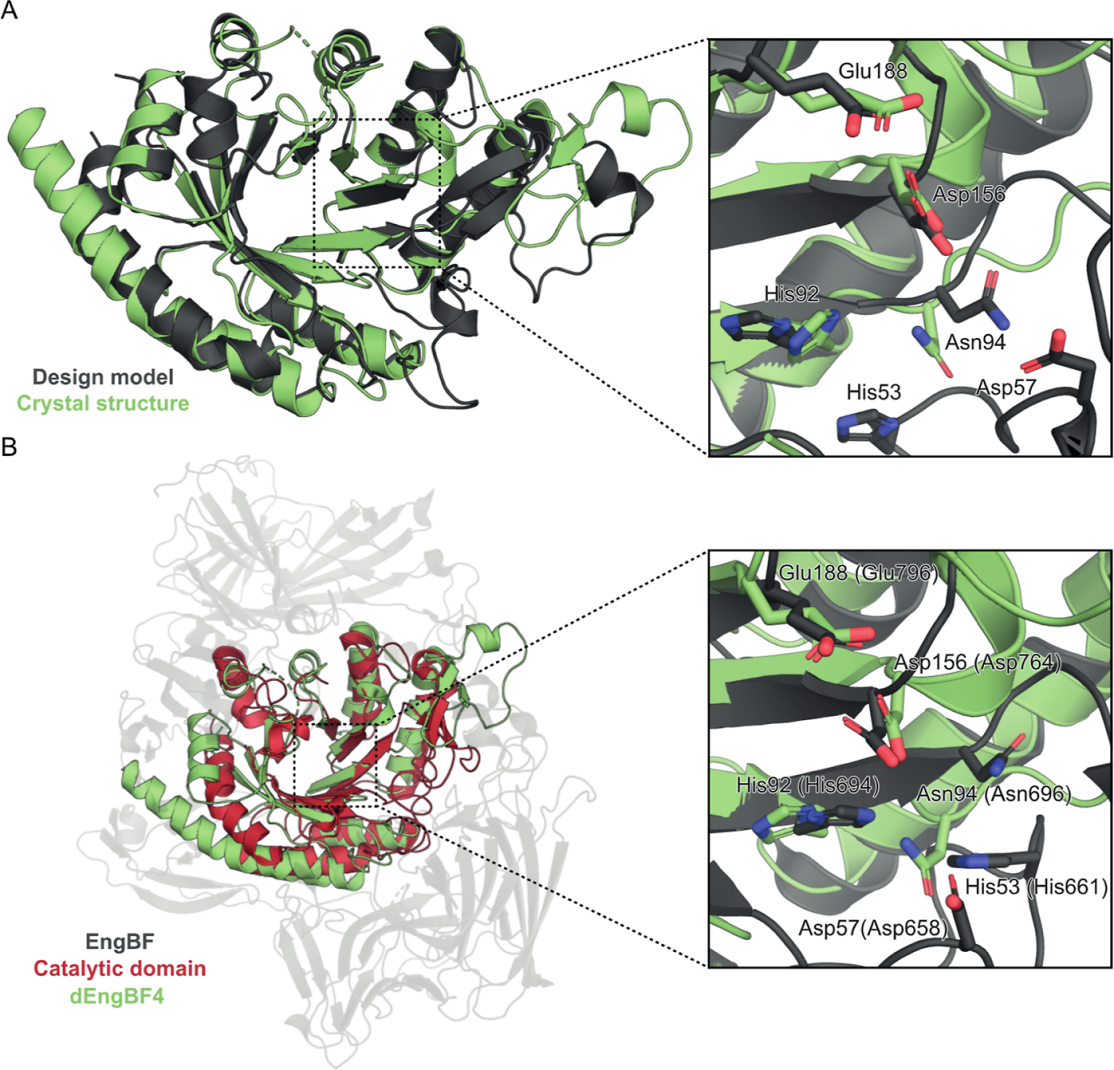
(A) Superposition of the dEngBF4 OmegaFold model (gray) and the crystal structure (green). The backbone Cα RMSD was 1.0 Å over 191 amino acids. The zoomed inset showcases catalytically essential amino acids, subject to both structural and sequence restraint in the design process. The side chain RMSD for catalytically relevant residues in the OmegaFold model and the defined in the crystal structure (Glu188, Asp156, His92, and Asn94) was 1.2 Å over 31 atoms. (B) Superposition of EngBF (PDB ID: 2ZXQ) (gray) with the restrained regions colored red and the dEngBF4 crystal structure (green). The backbone Cα RMSD was 2.6 Å for 115 amino acids. Zoomed insert depicts catalytically essential residues for which both structural and amino acid type restraint were applied in the design process. The side chain RMSD for catalytically relevant residues defined in the dEngBF4 crystal structure (Glu188, Asp156, His92, and Asn94) was 2.0 Å over 34 atoms. The EngBF numbering is indicated in parentheses.

The dEngBF4 crystal structure (form 1, molecule A) was aligned to the backbone structure of the designed model, revealing a Cα RMSD of 1.0 Å across 191 residues with respect to the OmegaFold model (Figure 8A). Notably, the native EngBF active site, which displayed an incomplete (β/α)_8_-barrel core, was successfully recapitulated in the design, now featuring a fully formed (β/α)_8_-barrel structure (Figure 8B). This led to a slightly higher overall backbone Cα RMSD of 2.6 Å across 115 residues when comparing the dEngBF4 crystal structure to that of the EngSP native enzyme (PDB ID: 5A55). The specifically restrained catalytic residues exhibited a Cα RMSD of 1.2 Å across 31 atoms relative to the OmegaFold model and 2.0 Å over 34 atoms relative to the EngBF enzyme crystal structure (Figure 8, inserts). Notably, His53 and Asp57 were excluded from this analysis due to their location within a loop region for which electron density was lacking (Figure 7A, insert I). Additionally, key catalytic residues, Glu188 and Asp156, corresponding to the catalytic acid/base and nucleophile, respectively, were found to be positioned within 3.4 Å, compared to the 4.2 Å separation observed in EngBF.

### Enzyme Candidates Do Not Display Substrate Binding or Measurable Enzymatic Activity

Enzymatic assays of the dEngBF proteins with Galβ(1–3)GalNAcα1-*para*-nitrophenol as a substrate showed no measurable *para*-nitrophenol release during the designated incubation period (data not shown). In addition, we probed the formation of a dEngBF-substrate complex using differential scanning calorimetry. However, no increase in the protein temperature stability in the presence of Galβ(1–3)GalNAcα1-*para*-nitrophenol was observed, indicating no interaction.

## Discussion

Based on the recent advances in deep network hallucination and protein structure prediction, a scaffold was designed to present the catalytic core of EngBF and reduce the size of the enzyme from about 130 to 30 kDa, while maintaining a stable and soluble protein. The potential of the approach is highlighted by the successful design of novel proteins that closely resemble the design target, exhibiting extremely high-yield expression in *E. coli* with monomeric structure and high melting temperatures.

Initial attempts at designing a reduced scaffold of EngBF using the publicly available trRosetta hallucination framework were hampered by either a poor reproduction of the active site structure or the formation of incompatible folds. Relatively simple modification of the hallucination loss function enabled the implementation of a weighted structural restraint map, which allowed for sufficient restraints to achieve a compatible scaffold with the correct active site geometry. Sequence space exploration was optimized in several ways, first by identifying favorable sequence features from short MCMC trajectories and second using these features to guide sequence modifications (Figure 1B). Notably, our findings demonstrate that trRosetta successfully restored the disrupted TIM-barrel-like fold as observed in the crystal structure of EngSP,^18^ resulting in the formation of a complete TIM-barrel structure in hallucinated designs.

While trRosetta was successful in hallucinating an overall protein scaffold, it is important to note that the network does not predict side chain conformations explicitly. Therefore, the resulting designs were generated solely on the basis of the backbone structure and did not consider specific side chain arrangements. This limitation is a potential obstacle when designing functional proteins, as precise positioning of side chains is often critical for proper enzyme function and protein interactions.^13^ In addition, the hallucinated designs did not yield high confidence scores from the best predictors, such as OmegaFold and RoseTTAFold. The implementation of the iterative ProteinMPNN sequence redesign led to the progressive refinement of the backbone through the OmegaFold models. This enhancement substantially boosted the prediction confidence, and our experimental results indicate that the ProteinMPNN designs from the later rounds exhibited superior solubility compared with those of the initial rounds. Furthermore, ProteinMPNN enabled exploration of sequence space inaccessible to trRosetta hallucination alone, improved the prediction confidence, and was broadly accessible without requiring customization of the design objective. While ProteinMPNN proved to be highly effective in improving the pLDDT confidence score, certain loop regions remained unrecoverable, which correlated with regions displaying poor or no electron density in the crystal structure. To improve the final design, integrating variable sequence lengths during the hallucination stage may fine-tune regions with a low prediction confidence. Alternatively, in a final refinement step, hallucinated designs could be further optimized by redesigning suboptimal loop regions (with low confidence scores or too high RMSFs) by adjusting their lengths and the two connected secondary structured elements. This would permit the introduction of novel structural elements or constrain loop dynamics, leading to a more refined scaffold architecture.

Although the success rate of designed proteins with attractive biophysical properties has improved, the dEngBF proteins characterized here exceed the size of the majority of de novo proteins.^47^ Furthermore, the proteins were designed to scaffold a complex enzyme active site, including long loops, which could potentially impact the overall stability. Remarkably, out of eight of our most refined candidates, six designs were readily expressed and adopted secondary structure elements consistent with those of the predicted structures (Figure 5B) including dynamic loops (Figure 7). The latter is quite remarkable given that de novo designed proteins often lack long and flexible loops^48,49^ and highlights the strength of hallucination for the generation of compact and stable scaffolds. However, while the presented design approach effectively produced small and stable scaffolds with an approximate arrangement of catalytic residues, two active site residues were located in highly dynamic regions. More accurate positioning of residues with catalytic properties needs to be addressed in future work, and when achieved the designed proteins can serve as an advantageous template for both random mutagenesis and rational protein engineering approaches. We envision two possible routes toward optimizing the active site structure. First, fine-tuning the backbone structure to enable more optimal placements of key catalytic residues, e.g., by combining RFdiffusion^50^ to sample structures around a promising design, such as dEngBF4, with RosettaMatch^51^ to select the most compatible backbones. This strategy could be combined with virtual docking of the substrate to different structural models in a competitive manner in an analogous approach as done with computational peptide affinity.^52^ Second, although enzyme dynamics is necessary for catalytic efficiency, some loop regions around the active site were probably too dynamic in our designs (e.g., lacking electron density in the crystal structure, showing high RMSF in Molecular Dynamics simulations, or having low pLDDT in predicted structures)—hindering accurate positioning of the catalytic residues or productive dynamics. Shortening or better structuring these loop regions (through loop modeling and adjustments on the connecting secondary structures) should improve the preorganization of active sites. Our approach indeed does not optimize the dynamic properties of the scaffold, which remains an outstanding challenge, and new computational methods need to be developed to properly optimize protein dynamics.

With the introduction of highly accurate structure prediction algorithms, the field of protein design has advanced significantly, now reaching a stage where practical applications in generating enzyme scaffolds for subsequent engineering are feasible.^14^ The success of such an approach has implications for how future enzyme engineering is addressed as it represents a general method that can contribute to designing functional proteins. To fully capitalize on the advantages of the approach presented in this work, future research will utilize the dEngBF proteins as templates for further protein engineering, incorporating the knowledge obtained here into the design phase to obtain enzymatic activity.

## Supporting Information Available

Average root-mean-square fluctuations of residues in the designed proteins and repeated DSC scans of dEngBF4 and dEngBF5 and protein sequences of the designed variants dEngBF1–11 (PDF)

## Accession Codes

Coordinates and structure factors have been deposited in the Protein Data Bank under accession codes 8QYE (form 1) and 8QZK (form 2).

## Abbreviations

EngSP: endo-α-*N*-acetylgalactosaminidases from *Streptococcus pneumoniae*
EngBF: endo-α-*N*-acetylgalactosaminidases from *Bifidobacterium longum*
RMSD: root-mean-square deviation
MCMC: Markov chain Monte Carlo
CD: circular dichroism
RMSF: root-mean-square fluctuations
