# Supplementary material for: Carving out a Glycoside Hydrolase Active Site for Incorporation into a New Protein Scaffold Using Deep Network Hallucination
Source: ACS Synth Biol. 2024 Feb 15;13(3):862–75. doi: 10.1021/acssynbio.3c00674 (PMC10949244; doi:10.1021/acssynbio.3c00674)
Supplement: Supplementary file 1 — sb3c00674_si_001.pdf [file sb3c00674_si_001.pdf]

Supporting Information

Carving Out A Glycoside Hydrolase Active Site For Incorporation Into A New Protein Scaffold Using Deep Network Hallucination

Anders Lønstrup Hansen, Frederik Friis Theisen, Ramon Crehuet, Enrique Marcos, Nushin Aghajari, Martin Willemoës

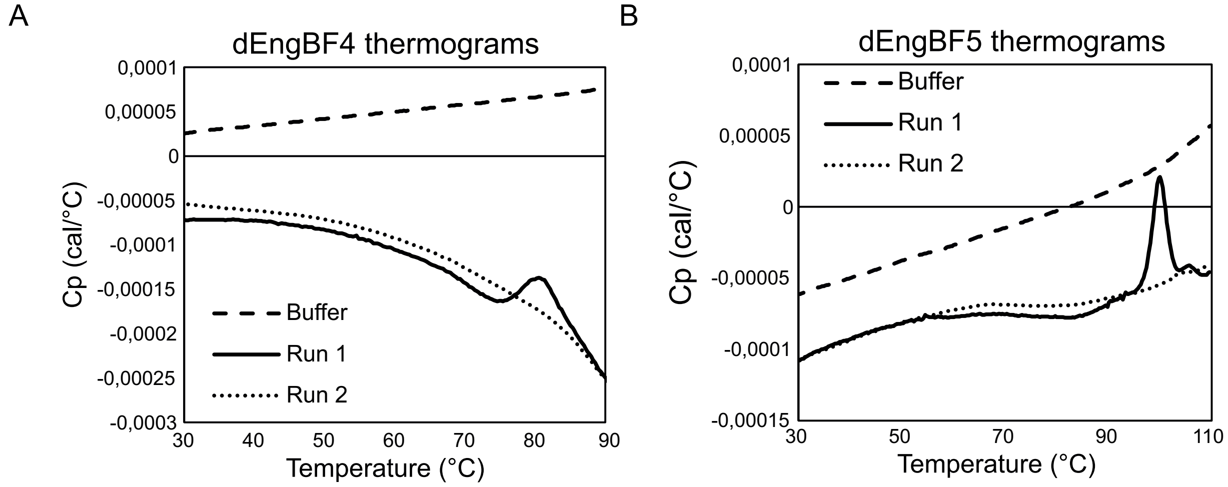

Supporting figure 1. DSC thermograms showing irreversible unfolding of dEngBF4 and dEngBF **5.** Differential scanning calorimetry thermograms of dEngBF4 (A) and dEngBF (B) unfolding experiment. The raw thermograms of buffer (dashed), first unfolding experiment (solid) and subsequent unfolding experiment (dotted) on the same sample are plotted as a function of temperature.

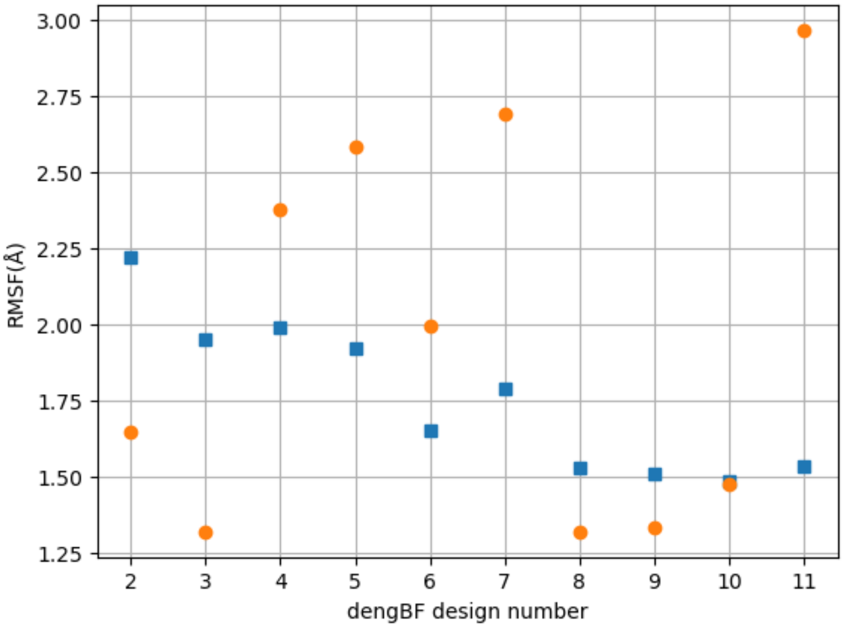

Supporting figure 2. Average Root Mean Square Fluctuations for all protein residues (blue squares) and 6 active site residues (orange circles) as reported by Theseus software for the eleven designed proteins. The 6 active site residues defined in the ProteinMPNN sequence redesign section are conserved in all designs and have the strongest structural restraints.

Supporting information table 1. Sequences of the designed dEngBF variants.

>dEngBF1
MEPIPDVHLMLTISPFGRRGHAPAEEAAQQAKLLRQAGPDHGILVHMKYWTGGGHDMFH
GGYAKEYAGQRTEAELKRIVRAYGNSNFIVFMHLNIHETPAVAGKTPEDFERHKRSWNWSG
QHKEIPLEQARDKKLPEALKRIERAMPATIVSFYVDVFMNTQIYEADPLDYRRLMEVAART
GIIVMFEWFKFYDKLLRSGTSVVFHWAISTAYISEDLVQIKDPELLKRNPGKVIVGVELNSFD
TLQKLIKNGVTSVALDVGGWQMFFNPRALRRLLRKIRKETVKRVLDGSGHHHHHH

> dEngBF2
MKKLPKVYFGLLIQAPGPDGGPSAEEQIELFEKVKKVGEGFGGLVFLDDPWGLHYDASHL
EFVPEDKGLRTLEEVKKIVDGAKEKDFYIAVHLNLTFMHPSDSFSEEELAAAPTFWNWSGE
NRAIPLEKLMEELFPKTLREIKEAFPDKLDWIHLDRLADGLVFELDEEEFRKIVREIKKLGLG
LMIESTKYLEVILEEGVEVIFFGALSKENLDGGLARAMDPRLYEKHPDRIILGVLAEDIETVE
RAIERGVKNLLLFLGGPEVTYNPEKIIEMIKKVKGELVEKALSGSGHHHHHH

> dEngBF3
MKKRPKVYFCLIIQAPGEDGGPTPEEVIKLFEEVKKVGEGIGGIVFLDDVWGEHYDSHHLD
WVPEARGLRTLEEVKRIVDAAKKLGFEIVVHLNLTFFSPRDSVSEEERKAAPPVWTWSGPL
RCIPLEKLINELFPKALEELREAFPGKLDWVYLDRIFDGDCFDLTPEQVRKILDWIKERGLG
VMIEDTRYLKTILEAGVKVIFNHALSEENFEEGKAKVLDPELYEKYPDQIIVGVKAESIETVE
KAIEKGVKNLAIELGGRNVTENIEKIKELIKKLSGELVKKALEGSGHHHHHH

> dEngBF4
MGEPAKIYINLGVQAPGPFGGGTPEEVAATLDRVKAAAPGIGIIVDLDEGSGVHRDSDHVRL
YPEGAGRYTLAEEQAIVDHAKALGAEICYHLNLTVFLPTDPVSEEERAAAKPVWTWTGLH
HCIPKEKLLETLLPQKLDELEAAFPGKLDYVYLDRLFDGRCYDLTDEEVRYVLDLIKERGL
GIKIESTKYLDTILESGVKVLVDEAVSEKNFDTGKAKVLDPKLFEKYPDLITVEVLYESIETIE
RALAAGVRNIAIHFGGYGVVSQLDEILDGVRAITENILALASAGSGHHHHHH

> dEngBF5
MAPAPAKIYLKIDVQGRSPRGGPTPEEVIAALERIKAAAPGIGIIVDLDHGWGAHFDSPHVAL
DPEGAGRYTLAELQTIVDAAKALGAKICIHLNLTVYLPTDPVSPEEIAAAPPIWTWAGLFHAI
PKDRVLNELLPEALARLDEAFPGKLDYVHLDHLFDGRAFDLSDEEVLYILNLIKDRGLGIKI
ESTKYLDVILDSGIKVLIDEAVSLETFDSGKAKVLDPELYKKYPDQIIVEVIAESVEVVRRAL
ERGVRNIAIDLGGYDVIAKLDENLAFVREVAEEIKALAAAGSGHHHHHH

> dEngBF6
MVELPEVTFQVYLQNPGPDGGPTVEQQIAEIAALKAEGAGWGLLLDIFFPDGRDFDMAHP
DYVDPEKGARTLEEIKAIVDGAKAQGFKISVHVNLTAMHPTDKFSEEEIAASEEFWTWLGK
RKKVPLKRLLETLFPETLAKIQAAFPGKVDYIDLDLLFTGEVYDLSDEQAREIVDLIKAKGL
GIKIESPRYLAIVLDAGVDVIYEGAWTPEAFPAGLGAALDPALYDRHPDRITLGLPAANLEM
VLRAIALGAKRLLLDLGGPDIRYDPAAKLALVKTVKARLEALAAAGSGHHHHHH

> dEngBF7
MARQLPEVTFEILLQNPGPDGGPTVEEQIALIGELKAAGAGYGLLLDIHLPDGRPYDMAHP
DYVPPEKGAYTLEQVQEIVDGAKAHGFKISVHINLTYMAPEDQFSEEELAAAPEFWTWLGK
RKKIPKDILLNRIFPETLERIQALFPGKIDYIELDLLFTGKVYDLSDEEFREIVERIKALGLGIEI
ESSKYLGIILDAGVKVLYEGAYTKENAPKGIGEALDPAYYDRHPDLITLGLNAADLEMVEK

AIDLGAKRLSLALTGAEIRYDPQAQLALVAEVKARLDALAAAGSGHHHHHH

> dEngBF8
MAMELPEVTFEVHLQHPTAAGGPTVAEQNAHLAALAAEAAGYGLLLDIHYQDGIYEDIAH
PSLVDPEKGPRTLEEIKAIVDAARAVGFKIKVHLNLVRMHPSMNFSEEEIANAEEFWTWSGK
RKKVPLERLLSELFPQALDDIEKAFPGKVDYIDLDELFSGKYYDLTDEQAQHIIDLIKARGL
GVEIENPRYLKIVLDAGVKVIFEGAYTPETFDAGLAAALDPELYDKYPDMITLALPAEDLAQ
ILEAIERGVKKLELDLSSPAILYDPAAQLALIRRVKATLEALAAAGSGHHHHHH

> dEngBF9
MHELPEVTFEIDLQHRTKDGGPTVEEQIAEIARLAEEAKGYGLLLDINFQDGVYEDIAHPDL
VDPELGPRTLEEIKAIVDGARAHGFKIKVHLNLVRMHPSDNFTAEEIAAAKEFWTWSGKRK
MTPLDRILNTLFPERLAAVQEAFPGQVDYIDLDELFSGKYYDLTEAQAKHIVALIKAQGLGV
EIESPKYLKIVLDAGVKVIFEGAYTPETYEEGLAAALDPALYDKHPDMITLALPATDLDMVF
DAIKKGAKKLKLRLDDPAIRYDPEARLALVREAKAALEALAAEGSGHHHHHH

>dEngBF10
MEGSGATLYVYLGVQPPGPFGGGTPEEVAAALDRVKAAAPDIGIIVDLDRAGELDPDSYHD
VFYPPGEGRYSEEEARAIVDHVKSLGGKIKFHLNLSVFHPTDPVSAEERAAAKPVWTWAGL
YHAVPKDRFLNEILPERLDKLEAAFPGKVDYVDLDLVFDGKCYDLTEEEVLYILQLIKDRGL
GIKIESDKYLDIILDSGVPVLYEGYVTAESLASGSNRALDPALFERYPDQLTVVVRYESVDVI
RRALELGVRRIAIHFGGNDLVGKLDRILAAVAEIVARILPLAAAGSGHHHHHH

> dEngBF11
MAPPPAEIYIYLGVQPPGPLGGGTPEEVKAALDKVKAAAPEIGIIVDLDDVGEIDPDSPHDVL
YPPGEGRYTEEEAREIVDHAKSLGAKINLHLNLAVYQPTDPASAEERAAGKPVWTWAGLF
HAVPKDRFLNTILPERLKKIEEAFPGKVDYVELDLVFSGKCYDLTDEEVKYILDLIKERGLGI
QIESSKYLDIILESGVPVLSAEFVTAESLASGSNRVLDPALFERHPDQITVVVLYESVDVIRQA
LALGVRRIAVHFGGSDIVGKLDKILAAVKEIVEKILPLARAGSGHHHHHH
